# Parenteral nutrition: Few more facts

**DOI:** 10.4103/0019-5049.65365

**Published:** 2010

**Authors:** Preety Mittal Roy, Vijaya Pant, Jyotirmoy Das

**Affiliations:** Department of Anaesthesiology, Pain and Perioperative Medicine, Fortis Hospital, Shalimar Bagh, New Delhi, India

Sir,

It was a pleasure reading the review article on parenteral nutrition (PN).[1] We congratulate the authors for their endeavor in making the topic so interesting. However, we feel that a couple of points need further discussion.

As per the guidelines for the provision and assessment of nutrition support therapy in adult critically ill patient set by the Society of Critical Care Medicine (SCCM) and American Society for Parenteral and Enteral Nutrition (A.S.P.E.N), 2009, serum protein markers (albumin, prealbumin, transferrin, C-reactive protein) are not recommended for determining adequacy of protein provision.[2] In all ICU patients receiving PN, initial mild ‘permissive underfeeding’ (providing approximately 80% of the total energy requirement) should be considered as it has been proved that excessive energy intake can lead to insulin resistance, greater infectious morbidity, extended mechanical ventilation and increased hospital length of stay. Eventually, as the patient stabilizes, PN may be increased to meet energy requirements.[2] The Canadian Critical Care Clinical Practice Guidelines’2003 states that there are insufficient supportive data to make a recommendation regarding parenteral Selenium supplementation in critically ill patients.[3] This has again been emphasized in the society’s 2009 guidelines.

Finally, in patients stabilized on PN, periodically repeated efforts should be made to initiate enteral nutrition (EN).[2] As tolerance improves and the volume of EN calories delivered increases, the amount of PN calories supplied should be reduced. PN should not be terminated, until ≥60% of target energy requirements are being delivered by the enteral route.
